# Unboxing cluster heatmaps

**DOI:** 10.1186/s12859-016-1442-6

**Published:** 2017-02-15

**Authors:** Sophie Engle, Sean Whalen, Alark Joshi, Katherine S. Pollard

**Affiliations:** 1 University of San Francisco, San Francisco, 94117 CA USA; 2 Gladstone Institutes, San Francisco, 94158 CA USA; 3 Division of Biostatistics, Institute for Human Genetics, and Institute for Computational Health Sciences, University of California, San Francisco, 94158 CA USA

**Keywords:** Systems biology/omics data, Bioinformatics visualization, Hierarchy data, Data clustering, Qualitative evaluation, Quantitative evaluation

## Abstract

**Background:**

Cluster heatmaps are commonly used in biology and related fields to reveal hierarchical clusters in data matrices. This visualization technique has high data density and reveal clusters better than unordered heatmaps alone. However, cluster heatmaps have known issues making them both time consuming to use and prone to error. We hypothesize that visualization techniques without the rigid grid constraint of cluster heatmaps will perform better at clustering-related tasks.

**Results:**

We developed an approach to “unbox” the heatmap values and embed them directly in the hierarchical clustering results, allowing us to use standard hierarchical visualization techniques as alternatives to cluster heatmaps. We then tested our hypothesis by conducting a survey of 45 practitioners to determine how cluster heatmaps are used, prototyping alternatives to cluster heatmaps using pair analytics with a computational biologist, and evaluating those alternatives with hour-long interviews of 5 practitioners and an Amazon Mechanical Turk user study with approximately 200 participants. We found statistically significant performance differences for most clustering-related tasks, and in the number of perceived visual clusters. Visit git.io/vw0t3 for our results.

**Conclusions:**

The optimal technique varied by task. However, gapmaps were preferred by the interviewed practitioners and outperformed or performed as well as cluster heatmaps for clustering-related tasks. Gapmaps are similar to cluster heatmaps, but relax the heatmap grid constraints by introducing gaps between rows and/or columns that are not closely clustered. Based on these results, we recommend users adopt gapmaps as an alternative to cluster heatmaps.

## Background

Cluster heatmaps are commonly used in biology and related fields to reveal hierarchical clusters in data matrices. Heatmaps visualize a data matrix by drawing a rectangular grid corresponding to rows and columns in the matrix, and coloring the cells by their values in the data matrix. In their most basic form, heatmaps have been used for over a century [1]. In addition to coloring cells, cluster heatmaps reorder the rows and/or columns of the matrix based on the results of hierarchical clustering. The hierarchical structure used to reorder the matrix is often displayed as dendrograms in the margins. Cluster heatmaps have high data density, allowing them to compact large amounts of information into a small space [2].

### Applications

Cluster heatmaps continue to find widespread application in biology [3–9]. They are most commonly used to visualize gene expression data across samples and conditions as measured by microarray or RNA-seq experiments. When applied to a correlation matrix, cluster heatmaps are particularly helpful at identifying groups of correlated samples or genes. These groups are revealed as block structures along the diagonal and can identify outliers, tissue subtypes, and novel gene pathways [10].

There are other applications of cluster heatmaps within biology beyond gene expression. Consider machine learning models trained on data where rows are samples and columns are predictors of a dependent variable such as a phenotype. Here, cluster heatmaps of correlation matrices are particularly helpful for identifying blocks of highly correlated samples that violate the independent and identically distributed (IID) assumptions made by most machine learning algorithms. They can also identify blocks of redundant predictors that may reduce predictive performance, increase computation time, or introduce collinearities that interfere with certain modeling techniques.

Finally, cluster heatmaps can help visualize the relationships between top predictive features, particularly when using estimates such as ensemble feature importances that lack a “directionality” that is more traditionally found in the positive or negative sign of linear model coefficients. If the relevance of a single feature to the positive or negative class is known, other features in the same block structure are likely relevant to the same class. Such applications are useful for interpreting “black-box” machine learning models, even for symmetric matrices of relatively small size.

### Shortcomings

Cluster heatmaps have several shortcomings [2, 11]. The Gestalt principles of proximity and similarity help define what clusters are visible in a heatmap; clusters are formed by cells that are close in proximity and visually similar in color [12]. However, the grid structure of the heatmap constrains how proximity may be used—we are limited to reordering the rows and columns of the heatmap. Thus, clusters may be perceived differently in the heatmap versus the dendrogram.

Flipping the right and left children in the dendrogram has no impact on the underlying data matrix or hierarchy, but has a major impact on how clusters are perceived in the heatmap. An optimal ordering can be found based on different metrics to place the most relevant rows or columns next to each other [13–15]. Even in that case, when clusters are formed close to the root of the dendrogram, cells that are not closely clustered must still be placed adjacent in the heatmap due to the rigid grid structure. Rows or columns that are closely clustered can also end up non-adjacent in large clusters.

To compensate, users must reference the dendrograms in the margins to be certain that visible clusters in the heatmap match the hierarchical clustering depicted in the dendrograms. It can be fatiguing and error-prone to shift focus back and forth between elements. These problems are particularly acute for large datasets where cluster heatmaps have even greater potential as a tool for data analysis.

### Alternatives

**Fig. 1:**
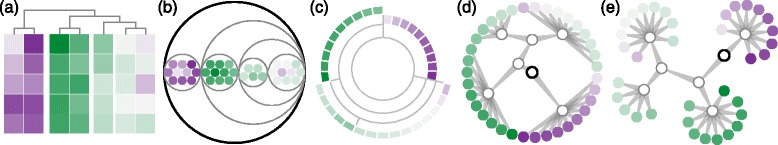
Alternatives to cluster heatmaps. We used these 5 alternatives (in addition to cluster heatmaps) in our final study. All five alternatives depicted here are for the same dataset. From *left* to *right*: (**a**) gapmap [11, 17] (**b**) circle packing [24], (**c**) sunburst [21], (**d**) radial dendrogram, and (**e**) force-directed tree [20]. Leaf nodes are filled to indicate the original value from the data matrix using the PRGn ColorBrewer scheme [66]. Positive values are *green in color*, and negative values are *purple in color*. The root node, if depicted, is indicated by a *black outline*. Inner nodes have *no fill color* and a *gray outline*. Edge tapering is used to indicate parent-child relationships in the node-link diagrams [37]

This work examines several standard hierarchical visualization techniques as alternatives to cluster heatmaps, as depicted in Fig. 1. Heer et al. [16] provides an excellent description of these techniques.

To review, *cluster heatmaps* visualize a hierarchically clustered data matrix using a reordered heatmap with dendrograms in the margin. *Gapmaps* [11, 17] are a recent variant of cluster heatmaps that encode the distance between the clusters as gaps between rows and/or columns. Both of these are juxtaposed techniques [18], combining heatmaps with dendrograms.

*Dendrograms* are a form of node-link diagram, where all of the leaf nodes are placed at the same level in the visualization. For traditional Cartesian dendrograms, this usually means the root node is at the top of the visualization and leaf nodes are found at the bottom. In a radial dendrogram, which uses polar instead of Cartesian coordinates, the root is in the center of a circle and the leaf nodes are arranged along the outer-most ring. For small datasets, radial layouts tend to use space more compactly than Cartesian layouts that often require considerable horizontal space [16].

Most node-link diagrams only differ in how the node layout is calculated. For example, *Reingold-Tilford trees* [19] do not place the leaf nodes at the same level. Instead, node placement corresponds more directly to the depth of that node in the tree. There are both rectangular/Cartesian and radial/polar versions with similar properties as dendrograms. *Force-directed trees* [20] use an approximation of a physics simulation to calculate node placement, where disconnected nodes repel each other and connected nodes attract each other. This results in a compact node-link diagram.

Space-filling techniques are an alternative to node-link diagrams that attempt to maximize (or fill) the display space used. *Sunbursts* [21] are space-filling adjacency diagrams very similar to radial Reingold-Tilford trees, except all nodes are represented by space-filling arcs radiating from the center of the visualization instead of individual circles. The root is encoded in the center, inner nodes are represented as nested arcs radiating away from the center, and leaf nodes are along the outermost rings of the circle. There is also a Cartesian variant sometimes referred to as partition or icicle diagrams.

In addition to space-filling adjacency diagrams, there are also space-filling enclosure diagrams that use nested shapes to encode hierarchy. The most common are *treemaps* [22], which use nested rectangles to depict hierarchy and the area of those rectangles to encode other values. *Squarified treemaps* [23] attempt to produce approximately square rectangles. While treemaps maximize the amount of space given to leaf nodes, the underlying hierarchy can be difficult to interpret. *Circle packing* [24] represents hierarchy via nested circles instead of squares, with the outermost circle representing the root and the innermost nested circles representing leaves. The tradeoff is less space dedicated to the leaf nodes, but often results in a clearer depiction of the hierarchy than treemaps.

### Contributions

We hypothesize that techniques without the rigid grid constraint of cluster heatmaps will perform better at clustering-related tasks when visualizing the results of hierarchical clustering. We test this hypothesis through a series of qualitative and quantitative user studies:

- *Practitioner survey:* We surveyed 45 practitioners in biology or related fields to understand how they use cluster heatmaps and determine the scope of experiments that would be useful to these practitioners.
- *Practitioner interviews:* We interviewed 5 practitioners to qualitatively evaluate our prototypes (see Fig. 1) and make adjustments prior to running a larger scale user study. Practitioners answered questions on each visualization technique and gave free-form feedback over an hour.
- *Mechanical Turk user study:* We finally conducted a between-subject Amazon Mechanical Turk user study for 6 visualization techniques. We had approximately 200 participants total, with over 30 participants per technique.

In addition to the above user studies, our contributions include the following:

- *Data processing:* We embedded the data matrix directly into the results of hierarchical clustering, enabling us to use standard hierarchical visualization techniques on this data.
- *Pair analytics:* We used a pair analytics pattern [25] with a domain expert in computational biology to identify and prototype alternatives to cluster heatmaps.

We found that no single technique was optimal for all tasks. However, gapmaps outperformed or performed as well as cluster heatmaps for clustering-related tasks. Given this technique was also preferred by our interviewed practitioners and can support large datasets, gapmaps are a promising alternative to cluster heatmaps. We discuss our findings in more detail in the following sections.

### Related work

Cluster heatmaps are widely used in biological applications such as genome-wide association studies [3, 7], genomic segmentation [4], exploring relationships between environmental variables and microbial communities [6], identifying patterns between signs and symptoms of chest pain [26], and others. Many implementations exist, including Bioconductor packages in R [27], the seaborn package in Python [28], stand-alone tools such as Cytoscape [29], GENE-E [30], Maple Tree, and Java Treeview [31], and web-based implementations [32, 33]. Gehlenborg and Wong have discussed the problems of using cluster heatmaps and discussed advantages of using gap maps as well as parallel coordinates [11].

Novel tools such as Furby [8, 34], OmicCircos [9], and QCanvas [5] have been recently presented for visualizing hierarchical data. Furby is a tool that allows interactive exploration of hierarchical clusters for biological applications [8]. They conducted preliminary evaluations with an expert user and found that for a force-directed layout, “a stable layout is preferred over an optimal one which takes longer to be created.” OmicCircos is a R-package that arranges heatmaps in a radial layout to visualize patterns [9]. Radial layouts have reduced performance compared to orthogonal layouts [35, 36]. QCanvas allows users to explore large-scale omics data, but information about its adoption is not reported [5].

Evaluating graph visualizations is important to understanding the strengths and weaknesses of graphical representations of hierarchical or network structures. Holten and van Wijk evaluated six different representations to reduce visual clutter in directed graphs [37]. Stasko et al. evaluated treemaps and sunburst representation methods to visualize hierarchical structures and found that users were faster and more accurate when using the sunburst representation for large graphs [21]. Kobsa conducted a user evaluation that evaluated frequently used tree visualization techniques [38]. Heer and Bostock used Amazon Mechanical Turk to evaluate design aspects such as chart size and gridline spacing for visual representations [39].

Lee et al. [40] introduced a graph-specific task taxonomy that extended Amar and Stasko’s [41] original task taxonomy. Saket et al. expanded the task taxonomy for evaluating graph representations that specialize in visualizing groups [42]. Their task taxonomy contained 31 total tasks that belonged to one of four task groups: group-only tasks, group-node tasks, group-link tasks, and group-network tasks. The tasks for our expert interviews and non-expert user study were drawn from a subset of their tasks.

Diehl et al. evaluated the benefits of radial representations and found although participants took less time when using cartesian layouts, the radial layout was useful for seeing trends in a single dimension [36]. Eye tracking was used by Burch et al. to evaluate radial, orthogonal, and traditional tree representations [35]. They found that participants performed poorly when using radial layouts as compared to both orthogonal and traditional tree layouts.

Treemaps are one of the most popular technique for visualizing hierarchical data [23, 43]. Novel techniques such as circle packing have been used for visualizing hierarchies [24, 44]. Ghoniem et al. compared node-link and matrix-based representations of graphs for readability [45]. Based on their evaluations, they found that participants performed poorly on path finding tasks when using matrix-based representations.

Jianu et al. used eye tracking-based evaluation to compare recent graph visualization techniques that include a semantic layer of set membership [46]. The techniques they evaluated were BubbleSets [47], LineSets [48], and GMaps [49]. BubbleSets and LineSets performed better than variations of GMaps and traditional node-link diagrams with colored nodes.

## Methods

We now present details regarding how we processed the data, developed prototypes, and conducted our qualitative and quantitative user studies. Please see git.io/vw0t3 for the raw data files from our practitioner survey and large-scale non-expert user study.

### Data processing

The process of running hierarchical clustering on a data matrix usually produces two separate but related datasets: a data matrix that has been reordered based on the clustering results, and trees representing the hierarchical clustering results. A cluster heatmap visualizes the reordered data matrix with a heatmap and the trees separately as dendrograms in the margins.

**Fig. 2:**
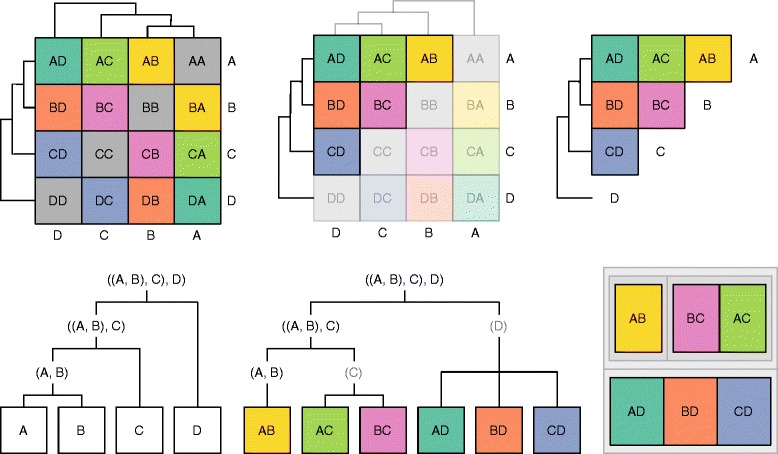
Unboxing Approach. Illustrates how the data matrix is “unboxed” and embedded into the hierarchical clustering of a symmetric matrix. The process is similar for asymmetric matrices, except there are no redundant cells to remove. *Top Left:* A standard cluster heatmap of a correlation matrix. *Top Middle:* The cluster heatmap with non-redundant information highlighted. *Top Right:* The cluster heatmap without the redundant information. *Bottom Left:* Hierarchical clustering of the variables (*rows* and *columns*) from the cluster heatmap, shown as a dendrogram. *Bottom Middle:* Hierarchical clustering of values (individual cells) and variables, shown as a dendrogram. *Bottom Right:* Hierarchical clustering of values and variables, shown as a treemap

Before exploring alternative visualization techniques for this data, we “unbox” the reordered data matrix and embed the cell values directly into the hierarchical clustering results. This is a key step that allows us to use standard hierarchical visualization techniques on the datasets underlying cluster heatmaps. At a high level, this process replaces leaves in the clustering tree that represented a row or column from the original data matrix with nodes for each appropriate cell instead. This results in a unified tree that contains both the hierarchical clustering results and the individual cell values from the data matrix. See Fig. 2 for an simplification of this process for symmetric matrices.

The full algorithm works for both symmetric and asymmetric matrices, and for clustering along one or both dimensions. We start by extracting the hierarchical clustering tree for rows. In that tree, each leaf currently represents a single row. We then add a copy of the column tree to each row leaf as a subtree. If the columns are not clustered (as in the case with symmetric matrices), we add one node per column to each row leaf instead. This gives us our nested clustering tree. Each leaf in the clustering tree is then resolved to its associated row and column, and replaced with the associated cell in the data matrix. Finally, we trim redundant nodes from the tree. If we have a symmetric correlation matrix, this involves removing the cells along the diagonal and in the lower triangle. We also remove unnecessary hierarchy during this step. For example, inner nodes with a single child node are replaced with that child such that *A*→*B*→*C* becomes *A*→*C* instead. This helps reduce the space requirements to visualize this hierarchy later. If we instead wanted to start with the column tree, we first transpose the matrix and then follow the same algorithm.

We applied this process to training data prepared for predictive modeling of the gene targets of distal enhancers in the K562 myelogenous leukemia human cell line. The original data consists of 399 features derived from chromatin immunoprecipitation followed by sequencing (ChIP-seq) and methylation assays generated by the Encyclopedia of DNA Elements (ENCODE) project [50]. Specifically, we processed the correlation matrices containing the top 16, 32, 64, 128, and 256 most important variables, as well as the full dataset of 399 variables. The processing of this data was implemented in R to match the existing analysis being done. We started with the hierarchical clustering results from the heatmap.2 function in the gplots package [51], transformed that output with the dplyr and reshape2 packages [52, 53], and exported the unified tree as a JSON file using the RJSONIO package [54].

We also generated a synthetic asymmetric dataset using the scikit-learn package in Python [55]. This allows us to control the specific numbers of samples, features, and clustering structure using an approach adapted from Guyon [56] for the Neural Information Processing Systems 2003 variable selection benchmark. To create evaluation datasets that balance both structure and noise, we used 4 informative features to generate both an 8 by 16 dataset (128 cells) and an 32 by 64 dataset (2048 cells). For both synthetic datasets, we reordered the columns of the data matrix using hierarchical clustering with complete linkage and an Euclidean distance metric using the SciPy Python package [57]. We applied our transformation using the NetworkX Python package [58] on the hierarchical clustering of both rows and columns.

At the end of this process, we had datasets that were both real and synthetic, symmetric and asymmetric, and with 100 cells or more. We used these datasets in our pair analytics development, practitioner interviews, and Amazon Mechanical Turk user study.

### Pair analytics

We used the pair analytics pattern [25] to iteratively develop and evaluate alternative visualization techniques to cluster heatmaps. This involved rapidly deploying prototypes and collecting feedback from a computational biologist at the Gladstone Institutes, who used the prototypes on the transformed ENCODE datasets to gain insights. This dataset was part of an active research project at that time.

We started with hierarchical visualization techniques capable of encoding the same data as a cluster heatmap. We focused on those techniques that had existing implementations in R [59] or Cytoscape [29]—two of the most used tools from our practitioner survey. We also wanted a mix of juxtaposed techniques [18], space-filling techniques, and node-link diagrams. We identified several possible alternatives based on this criteria: cluster heatmaps [1], gapmaps [11, 17], squarified treemaps [22, 23], partitions/icicles, sunbursts [21], circle packing [24], rectangular and circular dendrograms, rectangular and circular Reingold-Tilford trees [19], and force-directed trees [20].

We implemented our prototypes in D3 v3.0 [60] using the default implementations provided where possible. The prototypes supported both symmetric and asymmetric matrices, flexible dataset sizes, clustering along one or both dimensions, and limited interactivity via mouseover tooltips with row and column information for each node and the ability to make any tooltip “sticky” to serve as an annotation for important cells.

Based on the feedback from our domain expert and the minimum “useful” dataset size from our practitioner survey, we eliminated techniques that could not support datasets with at least 100 cells within 500 by 500 pixels. This would ensure that the visualization and question text would fit on most computer screens without scrolling for our large scale user study later. As a result, most rectangular/Cartesian layouts like Reingold-Tilford trees that traditionally take up a large amount of horizontal space were eliminated in favor of circular/polar/radial layouts that are more compact for small datasets [16].

Surprisingly, this also eliminated the space-filling treemap technique but not the space-filling circle packing technique. We found that the default squarified treemap algorithm could not consistently produce treemaps such that each leaf was large enough to interact with. Indeed, the algorithm is known to work poorly for balanced trees and when each leaf has equal size [23]. The domain expert also noted the importance of being able to determine which node was the parent versus child in the node-link diagrams. As a result, we used edge tapering to indicate parent-child relationships in the node-link diagrams [37]. The source node is indicated by a thick edge that tapers to a narrow point at the target node. We also made other minor modifications based on feedback.

**Fig. 3:**
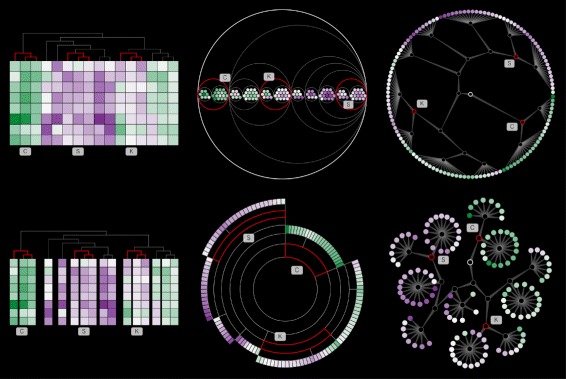
Mechanical Turk Example. Shows example images presented to Amazon Mechanical Turk participants for question 15 (see Table 1 for details). The question asked, “Which two of the highlighted elements are more closely clustered?” The correct answer is the pair K and S, since these clusters connect at a lower depth in the tree than cluster C

At the end of this pair analytics process, we identified 6 techniques for further user testing: cluster heatmap, gapmap, radial dendrogram, force-directed tree, sunburst, and circle packing. See Fig. 3 for examples of our implementations.

**Table 1:** Mechanical Turk user study analysis

Type	Task	Number	Question text	Nodes	Type	Mean	*χ* ^2^	df	*p*-value		
Training	1	1	Is the highlighted cluster mostly positive or mostly negative?	Clusters	N/A						
Training	1	2	What is the height of the tree?	N/A	N/A						
Timed	1	3	Which of the highlighted elements has the highest value?	Leaves	Score	0.380	4.578	5	4.695	E-01	
Timed	1	4	Is the highlighted cluster mostly positive or mostly negative?	Clusters	Score	0.661	14.650	5	1.197	E-02	∗
Timed	1	5	What is the height of the tree?	N/A	Error	3.083	23.278	5	2.987	E-04	∗∗∗
Training	2	6	Which of the highlighted elements is furthest away from the root?	Leaves	N/A						
Training	2	7	Which of the highlighted elements are siblings?	Leaves	N/A						
Timed	2	8	Which of the highlighted elements is furthest away from the root?	Leaves	Score	0.605	63.540	5	2.250	E-12	∗∗∗
Timed	2	9	Which of the highlighted elements is furthest away from the root?	Clusters	Score	0.732	14.705	5	1.170	E-02	∗
Timed	2	10	Which of the highlighted elements are siblings?	Clusters	Score	0.864	21.662	5	6.070	E-04	∗∗∗
Training	3	11	Which two of the highlighted elements are more closely clustered?	Clusters	N/A						
Training	3	12	How many visually distinct clusters do you see in this visualization?	N/A	N/A						
Timed	3	13	Which two of the highlighted elements are more closely clustered?	Siblings	Score	0.738	29.499	5	1.850	E-05	∗∗∗
Timed	3	14	Which two of the highlighted elements are more closely clustered?	Leaves	Score	0.275	12.775	5	2.558	E-02	∗
Timed	3	15	Which two of the highlighted elements are more closely clustered?	Clusters	Score	0.352	9.539	5	8.941	E-02	·
Timed	3	16	Which of the highlighted elements is least similar to its neighbors?	Clusters	Score	0.283	13.726	5	1.745	E-02	∗
Timed	3	17	How many visually distinct clusters do you see in this visualization?	N/A	Value	8.794	31.138	5	8.796	E-06	∗∗∗

**Fig. 4:**
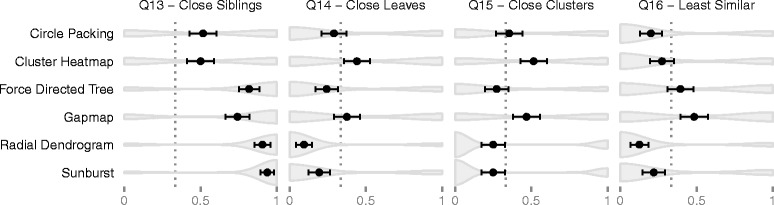
Mechanical Turk Average Scores. Shows the distribution of scores for clustering-related questions. The circle and black bar indicate the average score and standard error for each technique. The dotted line indicates random performance (not including the “Unsure” option). Our analysis shows that cluster heatmaps perform worse on average on question 13 than sunbursts, radial dendrograms, and force directed trees. Cluster heatmaps perform better on average than radial dendrograms in question 14. Question 15 did not have statistically significant differences in our post-hoc analysis. Gapmap performed better than radial dendrograms in question 16. Performance is worse than random for certain techniques in questions 14, 15, and 16—indicating their difficulty. However, there is at least one technique for each of these questions where the performance is better than random. See Tables 1 and 2 for more details

**Fig. 5:**
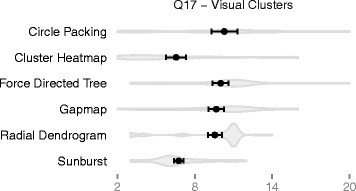
Mechanical Turk Average Clusters. Shows the distribution of responses to question 17, “How many visually distinct clusters do you see in this visualization?” The *circle* and *black bar* indicate the mean estimate and standard error for each technique. Our analysis shows that cluster heatmaps and sunbursts produce lower estimates on average compared to the other techniques. See Tables 1 and 2 for more details

Shows the type of question (training or timed), the task set, question number and text, the node type of the choices (leaf nodes, sibling nodes, or cluster nodes), the value type (score, absolute error, or raw value), overall average, and results (*χ* ^2^-test statistic, degrees of freedom, and *p*-value) from the per-question Kruskal-Wallis tests by technique. See Figs. 4 and 5 for the distribution of values for these questions broken down by technique

*Legend*: *** *p*≤ 0.001, ** *p*≤ 0.01, * *p*≤ 0.05, ^.^ *p*≤ 0.1

### Practitioner survey

The practitioner survey consisted of 15 questions, and was designed to take between 5 and 10 minutes to complete. Respondents had to be 18 years or older and have basic familiarity with cluster heatmaps to participate. We included questions on the background and experience of the practitioners, which visual elements they looked for in cluster heatmaps, the languages and tools they used to create and/or explore cluster heatmaps, and the types and sizes of data they typically visualized using cluster heatmaps.

We used an anonymous Google Form to collect responses over a 1 week period. To disseminate the form, we emailed the form to specific individuals and research labs that we knew had experience with cluster heatmaps and encouraged participants to forward the survey to others with relevant experience.

We had 48 total participants. There were 3 responses that did not pass the qualification checks and were filtered out of our remaining analysis. This left a total of 45 participants with valid responses. The overwhelming majority of practitioners held a doctorate degree, encountered heatmaps at least weekly, and had 5 years or more experience with cluster heatmaps. Areas of study included biology (e.g. cancer, developmental, molecular), genomics and genetics, and interdisciplinary fields such as biochemistry, biophysics, and bioinformatics.

We manually cleaned the long-form responses. This involved standardizing the text for the area of study, languages, tools, and dataset sizes entered by the users. For example, entries like “Java TreeView” and “Java tree view” were standardized to the text “Java TreeView” instead. Both the original and cleaned responses are available at git.io/vw0t3 online.

### Practitioner interviews

We collected responses from 5 academic biostatisticians at the Gladstone Institutes. Three had PhDs, one was nearing completion of a PhD, and one had a Master’s degree. Each participant had at least 5 years of experience using cluster heatmaps and primarily used the R language.

Interviews were conducted on-site to maximize familiarity. Participants were allowed to use their preferred web browser. Three participants accepted a $20 honorarium for an hour of their time, and two declined.

Participants evaluated 6 clustering techniques over the course of an hour. For each technique, they answered 4 questions to familiarize themselves with a particular technique via static images of the technique applied to an 8 by 16 synthetic dataset. These preliminary questions had correct answers, and the users were told the correct answer so that later answers were based on a better understanding of the technique.

They were then asked for free-form answers to subjective questions utilizing interactive plots of 8 by 16 and 32 by 64 synthetic datasets. These included “How many distinct clusters do you see in this visualization?,” “How would you summarize this dataset using this visualization?,” “Identify an interesting cluster and describe why it is interesting,” and “Did you find this visualization easy to interpret?” They were also asked about perceived advantages and disadvantages of each technique, and to rank and describe their 3 favorite techniques at the end.

### Mechanical Turk user study

We created a single Amazon Mechanical Turk Human Intelligence Task (HIT) with a maximum of 200 workers to test the accuracy and efficiency of our visualization prototypes. Amazon Mechanical Turk provides access to a diverse pool of participants with a wide range of age, ethnicity, and socio-economic status [39, 61]. Each worker was compensated $3.00 for completing the survey. Workers were not compensated for incomplete surveys. The maximum time limit was set to 20 minutes based on our pilots, although this was too short for a small subset of participants.

Participants were randomly redirected from Amazon Mechanical Turk to one of our six technique-specific surveys in Qualtrics. A total of 284 participants from Amazon Mechanical Turk completed our intake survey to confirm they were 18 years or older, but only 199 participants finished one of the technique-specific surveys. Between 32 to 34 users finished each technique survey. Examples of the surveys as well as the original and cleaned responses are at git.io/vw0t3 online.

#### Turk study design

We chose a between-subjects design such that each user participated in a single technique-specific survey, and users could not participate in multiple surveys. All of the technique-specific surveys had the same structure, questions, answers, order, and used the same datasets, but had different static images specific to the technique. All images had a maximum size of 500 by 500 pixels. Each survey began with basic tree definitions, and a brief description on how to interpret the visualization. This information was accessible in every question by clicking a “Toggle Help” button.

Each survey included three task sets, which were always presented in the same order. Each task set included 2 training questions and 3 to 5 timed questions. Participants were notified before training began, and were asked to focus on accuracy. If an incorrect answer was selected during training, a hint would show up above the “Next” button. Participants were also notified before the timing questions began, and asked to focus on both accuracy and efficiency for those questions. All questions were always presented in the same order and used forced-choice input via radio buttons or sliders. Participants had to select an answer to move to the next question, but could select “Unsure” if they were unable to answer a question. We collected browser information, timing information, and click information for all questions.

We began with questions that would help the participants understand how to interpret the visualization. The first task set focused on basics, including interpreting node color and the height of the tree. The second task set focused on hierarchy, including interpreting node distance from the root and whether nodes were siblings. Finally, the third task set focused on interpreting clusters. See Fig. 3 for an example clustering question from our study. Table 1 lists all of the questions asked for each task. Using the group level task taxonomy for graphs, our tasks fall under group-only (e.g. questions 3, 4), group-node (e.g. questions 8, 9, 10), and group-network tasks (e.g. questions 13, 14, 15) [42].

The maximum time limit and fixed compensation amount were the only mechanisms in place to ensure efficient responses. We also used participants with the “Masters” distinction, which requires those participants to consistently complete work with a high degree of accuracy. We did not implement any other formal engagement checks.

#### Turk study analysis

Incomplete surveys were not included in our analysis. We used the pandas package in Python [62] to combine survey responses and calculate the score for every response. We used the dplyr package in R for the remaining analysis [52]. We discarded all training questions and filtered out missing responses.

We filtered out spammers (whom want to complete the survey as quickly as possible) by removing participants with low-quality response patterns, responses completed too quickly for the user to actually participate in the task [61], and responses from participants that may have left open the survey for extended periods of time. First, we removed 3 participants that always selected the same choices. We then plotted the distribution of time it took to answer each question, and found the values ranged from 0 to 582 s. We conservatively chose to filter out a small number of responses that fell outside the 0.01 and 0.99 quantiles, which removed responses that took less than 3.8 s and greater than 74.3 s. The average time after filtering was 16 s per question. We did not run any additional analysis on the timing data.

We tested for statistically significant differences between techniques using ANOVA in R on logistic regression models for binary dependent variables such as score, and linear models for continuous dependent variables such as absolute error and raw value. We also ran a Kruskal-Wallis test since our data is not normally distributed. We ran these tests per question by technique. Both tests agreed on which questions had significant differences between techniques, although the exact level of significance differed slightly. These tests found statistically significant differences in the average scores of questions 13, 14, and 16, and in the values of question 17. We report the results from the Kruskal-Wallis test in Table 1.

Since the goal of task sets 1 and 2 was to familiarize the participants with the technique, we focused the remaining of our analysis on the clustering-related questions in task set 3. We performed a post-hoc analysis using Tukey’s HSD test for the scores of questions 13 through 16, and the value of question 17. The results of this analysis are provided in Table 2.

**Table 2:** Mechanical Turk post hoc analysis

	Q13				Q14					Q15					Q16					Q17					
Technique Pairs	est	err	*t*	*p*		est	err	*t*	*p*		est	err	*t*	*p*		est	err	*t*	*p*		est	err	*t*	*p*	
Cluster Heatmap, Gapmap	1.056	0.542	1.949	0.361		–0.274	0.503	–0.546	0.994		–0.186	0.497	–0.374	0.999		0.916	0.531	1.726	0.511		3.121	0.945	3.303	0.014	∗
Cluster Heatmap, Circle Packing	–0.065	0.504	–0.128	1.000		0.657	0.525	1.252	0.808		0.658	0.512	1.286	0.792		0.405	0.601	0.675	0.984		–3.745	0.960	–3.901	0.002	∗∗
Cluster Heatmap, Sunburst	2.708	0.811	3.338	0.010	∗	–1.191	0.571	–2.086	0.290		–1.159	0.537	–2.160	0.256		–0.292	0.579	–0.504	0.996		0.205	0.952	0.215	1.000	
Cluster Heatmap, Radial Dendrogram	2.269	0.702	3.232	0.015	∗	–2.032	0.698	–2.912	0.041	∗	–1.159	0.537	–2.160	0.256		–0.965	0.662	–1.457	0.688		3.003	0.960	3.128	0.025	∗
Cluster Heatmap, Force Directed Tree	1.504	0.573	2.623	0.087	·	–0.903	0.533	–1.694	0.530		–1.041	0.524	–1.989	0.348		0.550	0.529	1.040	0.903		3.455	0.938	3.683	0.004	∗∗
Gapmap, Circle Packing	0.992	0.546	1.817	0.443		0.383	0.538	0.711	0.980		0.473	0.516	0.916	0.942		1.322	0.581	2.275	0.202		–0.624	0.960	–0.650	0.987	
Gapmap, Sunburst	1.652	0.838	1.972	0.347		–0.916	0.583	–1.571	0.612		–0.973	0.541	–1.801	0.464		–1.208	0.559	–2.163	0.252		–2.917	0.952	–3.063	0.030	∗
Gapmap, Radial Dendrogram	1.213	0.732	1.656	0.550		–1.758	0.708	–2.483	0.126		–0.973	0.541	–1.801	0.464		–1.881	0.644	–2.921	0.040	∗	–0.118	0.960	–0.123	1.000	
Gapmap, Force Directed Tree	–0.448	0.610	–0.734	0.977		0.629	0.546	1.151	0.857		0.856	0.528	1.622	0.583		0.366	0.506	0.724	0.979		–0.333	0.938	–0.355	0.999	
Circle Packing, Sunburst	2.644	0.814	3.248	0.014	∗	–0.533	0.603	–0.885	0.949		–0.501	0.555	–0.903	0.946		0.113	0.625	0.181	1.000		–3.540	0.967	–3.660	0.004	∗∗
Circle Packing, Radial Dendrogram	2.204	0.705	3.127	0.021	∗	–1.375	0.724	–1.899	0.397		–0.501	0.555	–0.903	0.946		–0.560	0.703	–0.796	0.968		–0.742	0.975	–0.761	0.974	
Circle Packing, Force Directed Tree	1.440	0.577	2.495	0.119		–0.246	0.567	–0.433	0.998		–0.383	0.542	–0.707	0.981		0.956	0.579	1.650	0.561		–0.290	0.953	–0.305	1.000	
Sunburst, Radial Dendrogram	0.439	0.949	0.463	0.997		0.842	0.758	1.110	0.875		0.000	0.577	0.000	1.000		0.673	0.685	0.983	0.922		–2.798	0.967	–2.893	0.048	∗
Sunburst, Force Directed Tree	1.204	0.859	1.402	0.716		–0.288	0.610	–0.472	0.997		–0.118	0.565	–0.208	1.000		–0.842	0.557	–1.513	0.653		–3.250	0.945	–3.438	0.009	∗∗
Force Directed Tree, Radial Dendrogram	0.765	0.756	1.011	0.910		–1.129	0.730	–1.547	0.628		–0.118	0.565	–0.208	1.000		–1.515	0.642	–2.359	0.169		–0.452	0.953	–0.474	0.997	

Shows the significance results (estimate, standard error, *t*-value, and *p*-value) from running a post-hoc analysis using Tukey’s HSD test on clustering-related questions. There are several statistically significant differences in means for questions 13 and 17. The differences between the best and worst performers in questions 14 and 16 are also significant

*Legend*: *** *p*≤ 0.001, ** *p*≤ 0.01, * *p*≤ 0.05, ^.^ *p*≤ 0.1

## Results

We conducted several qualitative and quantitative studies to test whether hierarchical visualization techniques without the rigid grid constraint of cluster heatmaps perform better at clustering-related tasks. We discuss these results next.

### Practitioner survey

We conducted a survey of 45 practitioners in biology, genetics, and other related fields to learn more about how cluster heatmaps are used and determine the scope of experiments that would be useful to these practitioners. Visit git.io/vw0t3 for the survey results.

#### Survey results

We asked participants rate how often they viewed different visual elements in cluster heatmaps and used symmetric versus asymmetric matrices on a scale of 1 (never) to 5 (always). We then looked at the average response value. Practitioners most frequently looked for blocks of cells or bands of rows and/or columns in the heatmap (*a* *v* *g*=4.6,4.4 respectively). Practitioners also frequently looked for clusters in the dendrograms in the margins (*a* *v* *g*=4.1). Practitioners looked at the overview provided by the dendrogram and heatmap with less frequency (*a* *v* *g*=3.7,3.8 respectively). Most practitioners did not frequently look at the values of individual cells in the heatmap (*a* *v* *g*=2.6). They visualized both symmetric and asymmetric matrices with similar frequency (*a* *v* *g*=3.7,3.3).

We also looked at how many responses reported using different tools. The practitioners primarily used R (90%) and Cytoscape (80%) to generate cluster heatmaps. The dataset sizes reported varied widely. The median sizes ranged from approximately 100 to 250,000 cells (10 by 10 or 100 by 1000), but the variance was large. Some practitioners worked with datasets having 30,000 rows and/or columns.

#### Survey conclusions

We used the survey to make the simplifications necessary for a large scale non-expert user study while obtaining results that would still apply to expert practitioners.

Our first observation is that most practitioners are looking for adjacent blocks of rows and/or columns, confirming the importance of proximity in interpreting the hierarchical clustering results. Most practitioners also frequently reference the dendrograms of the cluster heatmap—further motivating our focus on hierarchical visualization techniques that are able to show the same information without the strict grid constraints of cluster heatmaps.

Additionally, most practitioners use R packages to generate static cluster heatmaps. This motivates our decision *not* to develop prototypes with robust interactivity until we have identified the most promising alternative techniques. We also decided to focus on those techniques available in the tools frequently used by practitioners to increase the potential for wide-scale adoption. Finally, we conclude that our datasets must have at least 100 cells or more to be useful for practitioners. However, the matrices may be either symmetric or asymmetric.

### Practitioner interviews

After conducting the practitioner survey, we used pair analytics to develop several visualization alternatives to cluster heatmaps. We conducted 1 h interviews with 5 academic biostatisticians to pilot these alternatives, and used their feedback to inform the design of our Amazon Mechanical Turk study.

#### Interview results

Participants were shown several synthetic asymmetric matrices and asked their preferences. They preferred traditional cluster heatmaps as well as gapmaps, with no clear third preference. Gapmaps were preferred by all but one participant, who found the spacing distracting when quickly scanning for patterns and anticipated the gaps would interfere with the metadata commonly plotted along the axes.

Other alternates were heavily criticized for losing the row structure of asymmetric matrices. This structure is especially important to determine which cells belong to the same sample. Practitioners found this loss of information so disorienting that it outweighed any advantages they identified in other techniques. Non-heatmaps were also criticized for their “architectural” or “design” qualities, related to a preference against visualizations that are popular but are often perceived as overly complex (e.g. the “ridiculogram” [63, 64]).

Rather than refer to the hierarchy illustrated by an unfamiliar visualization technique, practitioners were prone to use color to infer the hierarchy. One practitioner also pointed out the poor use of white as a background color in our prototypes.

Finally, practitioners also noted that cluster heatmaps often encode the linkage distance between clusters by varying the level height in the dendrograms. Our prototype instead used equal height for each level.

#### Interview conclusions

This study confirmed a heavy practitioner preference towards familiar techniques, namely cluster heatmaps and gapmaps. While alternate techniques were seen as helpful for certain tasks, their advantages did not outweigh their unfamiliarity and the loss of row/column information. Based on these results, we proceeded with a larger scale non-expert user study to quantitatively compare these techniques. We made several minor modifications to our prototypes including the use of a black background.

However, we were unable to encode linkage distance between clusters for every alternative technique. The interviewed practitioners also only evaluated these techniques for asymmetric matrices—which was their primary use case. Correlation matrices are symmetric and rely less on row and column information, and the loss of this context may be less of an issue. We revisit these issues in the “Discussion” section.

### Mechanical Turk user study

We conducted an Amazon Mechanical Turk user study with approximately 200 participants to evaluate how well different visualization techniques perform at clustering-related tasks. See Table 1 for a summary of the questions. Visit git.io/vw0t3 for the raw results.

#### Turk study results

We tested for statistically significant differences in accuracy across techniques. See the “Methods” section for details on our analysis, and Tables 1 and 2 for the results of this analysis. We found statistically significant differences in the average scores for clustering-related questions. These results are illustrated in Fig. 4.

Question 13 and 14 asked participants to estimate which pair of nodes were more closely clustered. Question 13 included two leaf nodes that were siblings, and question 14 did not. Our analysis showed that cluster heatmaps performed statistically significantly worse than most other techniques in question 13. The results were mixed for question 14; the only statistically significant finding was that cluster heatmaps performed better than radial dendrograms.

Question 15 asked users to estimate which pair of inner cluster nodes were more closely clustered. The differences were barely statistically significant, and no significance was found in our post-hoc analysis. Question 16 asked participants to determine which pair of nodes were least similar to their neighbors. Gapmaps outperform radial dendrograms, but none of the other differences were statistically significant.

We also compared whether the scores (including error bars) for questions 13 through 16 were better than random. Participants could choose between four values for those questions, but one of those values was an “unsure” option. Removing the “unsure” option from consideration, random performance is 1 out of 3. For question 13, the performance was better than random for all techniques. For question 14, performance was only better than random for cluster heatmap, with gapmap and circle packing falling on the threshold. For question 15, the performance of both cluster heatmap and gapmap were better than random. For question 16, only gapmap outperformed random, but force directed tree and cluster heatmap fall on the threshold.

Finally, question 17 asked participants to estimate the number of clusters from the visualization. This is a subjective question—there is no single correct answer. We found that cluster heatmaps produced significantly lower estimates on average compared to all other techniques except for sunbursts. See Fig. 5 for details.

#### Turk study conclusions

No single technique consistently ranked best at all clustering-related tasks. Cluster heatmaps was among the worst performers for question 13, among the best performers on questions 14 and 15, and had mediocre performance for question 16. Radial dendrograms and sunbursts are among the best performers on question 13, but are among the worst performers on questions 14–16. Force directed trees did moderately well on questions 13 and 16, but poorly on questions 14 and 15. The performance of circle packing was mediocre across all clustering questions.

The results show consistently high performance for gapmaps, even if not always top ranked. Gapmaps outperform cluster heatmaps on question 13, have performance similar to cluster heatmaps on questions 14 and 15, and are pulling ahead of cluster heatmaps on question 16. Given that this technique can also support dense datasets and were liked by our practitioners, gapmaps have significant promise as an alternative to cluster heatmaps.

A large caveat, however, is how poor the performance is overall. Only question 13 outperformed random chance for all techniques. Questions 14 through 16 only had 1 or 2 techniques that clearly outperformed random chance—indicating the difficulty of these questions for novice users. It would be interesting to compare these findings with a more expert audience that could achieve higher scores.

## Discussion

Involving practitioners at multiple stages in this project was critical. Thanks to feedback we received from practitioners via the survey, the pair analytics development pattern, and the one-on-one interviews, we were able to identify several cases where our assumptions did not necessarily hold.

For example, we anticipated the loss of row and/or column labels would be an issue for some techniques. However, we did not anticipate how important the context provided by the grid was for asymmetric matrices. For example, cells that belong to the same row often belong to the same sample. Depending on the dimensions being clustered, cells from the same row may become indistinguishable from those belonging to different rows in many of our alternatives. This is not an issue when visualizing symmetric correlation matrices where this context is not as informative, but symmetric matrices were a smaller subset of many practitioner’s common use cases. Given this, it is possible that the techniques that did not include this context may perform better in studies focused on symmetric matrices.

Symmetric matrices have other important advantages. Since half the cells are redundant, alternative techniques can utilize this space to better support larger datasets. Also, these matrices need only be clustered along a single dimension. This reduces the amount of nesting required to illustrate the hierarchy.

There are many other research directions to explore. There is still room for optimization of our “unboxing” approach to reduce unnecessary levels of nesting. This is especially important when both the rows and columns are clustered, which is common for asymmetric matrices.

We focused on techniques practitioners could immediately adopt via existing tools—but customized implementations and novel techniques may produce better results. We also used color to encode the value from the cell across all techniques to keep the encoding consistent with that of cluster heatmaps. However, area (commonly used in space-filling techniques) is more effective for encoding quantitative values [65]. Encoding linkage distance in these techniques is another potential direction of research.

Interactivity is critical to explore further as well, although it is difficult to user test interactivity on a large scale. Even a cluster heatmap is unable to display the entire dataset at a certain size. This makes critical the ability to search, sort, filter, and brush. In this interactive setting, the ease of navigation with rectangular layouts may begin to outweigh the compactness of some circular layouts.

Given these caveats and the results of our qualitative and quantitative studies, gapmaps are a promising alternative to cluster heatmaps for asymmetric matrices. Symmetric matrices may be suited to additional alternative techniques and need more study.

## Conclusions

Cluster heatmaps have become a staple of biological and biomedical research since their introduction in the field over 20 years ago [1], and are clearly a valuable visualization technique for these practitioners. However, while cluster heatmaps have high density, they suffer from issues caused by their rigid grid layout [2, 11].

Motivated by our own use and reinforced by a series of qualitative and quantitative user studies, we used pair analytics with a computational biologist to develop alternative visualization techniques based on “unboxing” heatmap cells and embedding them directly into hierarchical clustering results. By relaxing the grid constraint of cluster heatmaps, our unboxing approach aimed to improve performance of tasks where practitioners commonly shift their attention back and forth between the cells and the hierarchy. Such tasks are common for many, but not all, practitioners.

Our study involved practitioners from biology and related fields at multiple stages in our development and evaluation process. We surveyed 45 practitioners to learn how they use cluster heatmaps, and evaluated our alternatives via hour-long interviews with 5 practitioners and an Amazon Mechanical Turk user study with 200 participants.

While more study is needed, we found multiple statistically significant differences in average performance between several techniques. No single technique consistently ranked best at all clustering-related tasks. For example, radial dendrograms, force directed trees, and sunbursts were among the best performers at identifying closely clustered siblings, but performed poorly for longer-distance relationships. The performance of circle packing was mediocre for all clustering tasks. However, gapmaps either outperformed or performed as well as cluster heatmaps for clustering-related tasks.

Integrating these non-expert large-scale results with our smaller-scale expert interviews, we conclude that gapmaps are a promising alternative to cluster heatmaps for asymmetric matrices, while other hierarchical techniques may not improve performance enough to justify their adoption by practitioners for this use case. However, more exploration is needed for the specific case of symmetric matrices.

## Abbreviations

ANOVA: ANalysis Of VAriance
ENCODE: Encyclopedia of DNA elements
HIT: Human intelligence task
HSD: Honest significant difference
JSON: JavaScript object notation
